# Hepatitis B Reactivation During Ribociclib Therapy in a Patient with Metastatic Breast Cancer

**DOI:** 10.5152/tjg.2026.26186

**Published:** 2026-06-03

**Authors:** Yunus Emre Demiral, Yunus Günegül, Ahmet Tarık Eminler, Mukaddes Tozlu, Mustafa İhsan Uslan

**Affiliations:** Department of Gastroenterology, Sakarya University Faculty of Medicine, Sakarya, Türkiye

Dear Editor,

Hepatitis B virus (HBV) reactivation is a recognized complication of immunosuppressive therapy and may lead to acute hepatitis, liver failure, and interruption of anticancer treatment.^1^ Cyclin-dependent kinase 4/6 (CDK4/6) inhibitors, including ribociclib, are widely used in hormone receptor–positive metastatic breast cancer and are generally considered low risk for HBV reactivation. However, data regarding HBV reactivation in patients receiving CDK4/6 inhibitors remain limited.^2^ Herein, we report a case of HBV reactivation occurring during ribociclib therapy after the discontinuation of antiviral prophylaxis.

A 61-year-old woman was diagnosed with metastatic breast cancer in July 2023. Informed consent was obtained from the patient. Before the initiation of targeted oncologic therapy, HBV screening revealed anti-HBc immunoglobulin G (IgG) positivity, hepatitis B e antigen (HBeAg) negativity, anti-HBe positivity, and anti-HBs negativity. Hepatitis B surface antigen (HBsAg) was reported as borderline (index value within the 0.90-0.99 S/CO range); however, the concurrent presence of detectable baseline HBV DNA (12 copies/mL) confirmed a true low-level viremic, HBeAg-negative chronic HBV infection rather than a false-positive assay or a simple resolved infection. Liver function test results were normal. In accordance with guideline recommendations, prophylactic tenofovir disoproxil fumarate (TDF) was initiated concurrently with ribociclib therapy on March 1, 2024.

On January 23, 2025, she was reevaluated because of elevated liver enzymes. Upon detailed clinical inquiry, it was revealed that she had discontinued antiviral prophylaxis on October 30, 2024, without medical consultation. She stated that she stopped the medication because of polypharmacy fatigue and a misunderstanding regarding the need for continuous long-term antiviral therapy during oncologic treatment. She was asymptomatic and denied alcohol intake, herbal medication use, or new drug exposure. Ribociclib was the only ongoing anticancer therapy. Physical examination was unremarkable. Laboratory tests demonstrated alanine aminotransferase (ALT) 253 U/L, aspartate aminotransferase (AST) 387 U/L, and total bilirubin 4.02 mg/dL. HBV serology revealed HBsAg positivity and a marked increase in HBV DNA to 10^7^ copies/mL. A comprehensive workup for alternative etiologies, including other hepatotropic viruses (Hepatitis A virus, hepatitis C virus, and hepatitis E virus) and autoimmune hepatitis (negative antinuclear antibody, anti-smooth muscle antibody, and normal IgG levels), was unremarkable. Abdominal and portal vein Doppler ultrasonography were unremarkable.

HBV reactivation was diagnosed, and TDF therapy was reinitiated on January 24, 2025. One month after reinitiation of antiviral therapy, ALT and AST levels decreased to 175 U/L and 176 U/L, respectively, with a total bilirubin level of 1.16 mg/dL. By the fourth month, transaminase levels had normalized, and HBV DNA became undetectable.

HBV reactivation is well described in patients with prior HBV exposure receiving immunosuppressive therapy. The European Association for the Study of the Liver and the American Association for the Study of Liver Diseases recommend antiviral prophylaxis in HBsAg-positive patients and risk-based monitoring or prophylaxis in anti-HBc-positive individuals undergoing immunosuppressive treatment.^3^^-^^4^ Although certain conventional biologic therapies are associated with a very low risk of HBV reactivation in the HBsAg-negative phase,^5^ the risk associated with novel targeted therapies warrants careful consideration. This is especially critical in patients with complex viral profiles or indeterminate phases of infection, who remain highly vulnerable to HBV reactivation without adequate viral suppression.^6^ Although CDK4/6 inhibitors are generally considered to confer a low risk of HBV reactivation, experimental data suggest that CDK4/6 inhibition may affect T-cell proliferation and immune regulation. Given the central role of T-cell–mediated immunity in controlling HBV replication, these immunomodulatory effects could theoretically affect viral immune surveillance. However, the clinical relevance of this mechanism remains uncertain, and available evidence is limited.^7^

In the present case, the temporal association between the discontinuation of antiviral prophylaxis and the rapid increase in HBV DNA indicates that the loss of antiviral suppression may have been the primary trigger for reactivation. The patient’s baseline profile was consistent with an HBeAg-negative chronic HBV infection. According to current risk-stratification models, HBsAg-positive patients receiving targeted oncologic therapies, even in the presence of low-level viremia, are considered high risk (>10%) for HBV reactivation. This classification underscores that continuous antiviral prophylaxis was clinically mandatory in this patient. Although ribociclib may have contributed to a permissive immunomodulatory environment, clinical evidence supporting this mechanism remains limited. Therefore, this event is more consistent with HBV reactivation secondary to prophylaxis withdrawal rather than with direct drug-induced reactivation. Although ribociclib is independently associated with transaminase elevations as an adverse effect, the key differentiating factor in this case was the marked increase in HBV DNA levels to 10^7^ copies/mL, supporting HBV flare rather than isolated drug-induced hepatotoxicity.

A previously published report described the safe administration of ribociclib in a patient with HBV infection who remained on continuous antiviral therapy. In the present case, clinically significant HBV reactivation occurred after the interruption of antiviral prophylaxis during ribociclib treatment. Furthermore, a recent report described severe HBV reactivation and liver failure associated with dalpiciclib, another CDK4/6 inhibitor.^8^ These findings suggest that the safety profile of this drug class in patients with HBV infection warrants further investigation.

In conclusion, this case highlights HBV reactivation following the discontinuation of antiviral prophylaxis during ribociclib therapy. Although the independent contribution of CDK4/6 inhibition remains uncertain, this case adds to the limited clinical evidence regarding HBV reactivation in patients with prior HBV infection receiving targeted oncologic treatment.

## Data Availability

The data that support the findings of this study are available on request from the corresponding author.
